# A Biterm Topic Model for Sparse Mutation Data

**DOI:** 10.3390/cancers15051601

**Published:** 2023-03-04

**Authors:** Itay Sason, Yuexi Chen, Mark D. M. Leiserson, Roded Sharan

**Affiliations:** 1 School of Computer Science, Tel Aviv University, Tel Aviv 69978, Israel; 2 Department of Computer Science and Center for Bioinformatics and Computational Biology, University of Maryland, College Park, MD 20740, USA

**Keywords:** mutational signature, panel sequencing data, biterm topic model

## Abstract

**Simple Summary:**

We developed an efficient method for analyzing sparse mutation data based on mutation co-occurrence to infer the underlying numbers of mutational signatures and sample clusters that gave rise to the data.

**Abstract:**

Mutational signature analysis promises to reveal the processes that shape cancer genomes for applications in diagnosis and therapy. However, most current methods are geared toward rich mutation data that has been extracted from whole-genome or whole-exome sequencing. Methods that process sparse mutation data typically found in practice are only in the earliest stages of development. In particular, we previously developed the Mix model that clusters samples to handle data sparsity. However, the Mix model had two hyper-parameters, including the number of signatures and the number of clusters, that were very costly to learn. Therefore, we devised a new method that was several orders-of-magnitude more efficient for handling sparse data, was based on mutation co-occurrences, and imitated word co-occurrence analyses of Twitter texts. We showed that the model produced significantly improved hyper-parameter estimates that led to higher likelihoods of discovering overlooked data and had better correspondence with known signatures.

## 1. Introduction

Statistical models for discovering and characterizing mutational signatures are crucial for revealing biomarkers for practical applications. Mutational signatures reveal the mutational processes that transform a “normal” genome into a cancerous genome. The activity of these processes have provided insights into the development of tumorigenesis, and they also have led to new and expanded potential applications for personalized data [1]. Consequently, as more and more cancer data become available, significant efforts have been made to introduce statistical models that can accurately and effectively capture these signatures.

Most models of mutational signatures of cancer represent each *N* patient with cancer as having mutations that were generated from a linear combination of *K* mutational signatures. Therefore, each signature is represented as a probability distribution over a set of mutational categories, which are typically the 96 categories given by the 6 single base substitution types and the 5’ and 3’ flanking bases [2]. Each patient’s mutations are represented as exposures to the mutational signatures, in addition to some noise. Alexandrov et al. [2,3] were the first to use non-negative matrix factorization (NMF) to perform a census of mutation signatures across thousands of tumors. Subsequent methods have used different forms of NMF [4,5,6,7], or have focused on inferring the exposures (also known as refitting) based on the signatures and mutation counts [8,9,10]. More recent approaches have borrowed from the world of topic modeling in order to provide a probabilistic model of the data so as to maximize the model’s success [11,12,13,14]. The Catalogue of Somatic Mutations in Cancer (COSMIC) now includes a census of dozens of validated mutational signatures [2,15,16], (https://cancer.sanger.ac.uk/signatures/ accessed on 1 January 2022), and there have been many efforts to investigate using these signatures as biomarkers for diagnosis and known cancer therapies (e.g., [17,18,19]).

Developing methods for analyzing mutational signatures in targeted sequencing datasets have presented new opportunities in the research. To date, most efforts to model mutational signatures have focused on data-rich scenarios, such as whole-exome or whole-genome sequences, where there are from dozens to even thousands of mutations per patient. The most popular targeted sequencing panels have only included several hundred genes [20,21] and, in general, have had fewer than 10 mutations per patient [20,22]. The standard topic modeling and non-negative matrix factorization frameworks are not capable of generalizing according to such cases [19,23], even though targeted sequencing has been more common in clinical practice. Methods that could accurately infer exposures from targeted sequencing data were thus critical for demonstrating the potential of mutational signatures-based precision medicine in real applications [1,17]. At the same time, the largest targeted sequencing datasets have included data from many more samples (e.g., see [24]). Therefore, along with scaling and sparsity challenges, there is also an opportunity for discovering novel and rare signatures.

To partially address this challenge, SigMA [19] relied on whole-genome training data to interpret sparse samples and predict their homologous recombination deficiency status. However, SigMA still suffered from the fact that not all cancer types have available whole-genome sequencing data. The Mix model [25] simultaneously clustered the samples and learned the mutational landscape of each cluster, thereby overcoming the sparsity problem. However, it still suffered from high computational costs when learning its hyper-parameters.

Therefore, we developed a new topic model for sparse data that borrowed from similar works in the natural language processing (NLP) domain. Specifically, the advent of Twitter has produced an explosion of much shorter (sparser) documents that researchers have to analyzed, where “documents” have a mean length of <35 characters [26]. One of the main insights for handling sparse documents has been to model word co-occurrence directly [27], under the assumption that words that co-occur frequently were likely from the same topic. While computationally much more intensive than the standard topic model, co-occurrence has shown greater sensitivity on sparse datasets.

Following the biterm topic model [27], we proposed modeling mutation co-occurrence in a similar way. In detail, the generation of each mutation pair was modeled as a two-step process. First, a signature was chosen from a global, cohort-level exposure vector θ, and then a pair of mutations was drawn from that signature. The rationale was that, in the case of mutational signatures, the “vocabulary” (mutational categories) was much smaller than that of Twitter. In a targeted sequencing setting, only approximately 0.1% of a patient’s mutations can be observed. Therefore, modeling the co-occurrence of mutations could provide additional signals as the number of data points (i.e., mutation pairs) would be quadratic for the number of mutations. Furthermore, because the number of mutational categories was low, it would also be computationally feasible.

In the next section, we formally described the model and provided an expectation-maximization (EM) framework for learning the model parameters and estimating the number of signatures in the data. Then, we applied it to various simulated and real targeted sequencing datasets and showed that the model was significantly more efficient and outperformed other hyper-parameter estimation methods. This method was used as a pre-processing step for the Mix method, which improved the training time by an order of magnitude and led to higher likelihoods of discovering overlooked data and improving the correspondence with known signatures.

## 2. Materials and Methods

### 2.1. Preliminaries

We followed previously published research and assumed that the somatic mutations in cancer fell into M=96 categories (denoting the mutation identity and its flanking bases). These mutations were assumed to be the result of the activity of *K* (a hyper-parameter) mutational processes, each of which was associated with a signature $S_{i}=\left(e_{i}\left(1\right)\cdots e_{i}\left(M\right)\right)$ of probabilities to represent each of the mutation categories.For a given genome *n*, we denoted its mutation categories as $O^{n}=\left(o_{1}^{n}\cdots o_{T_{n}}^{n}\right)$ and assumed that this sequence was represented by the (hidden) signature sequence $Z^{n}=\left(z_{1}^{n}\cdots z_{T_{n}}^{n}\right)$. We denoted the exposures of the signatures across all patients as $\pi =(\pi_{1},\cdots ,\pi_{K})$. Note that, as compared to most previous works, this was a single “global” exposure vector, rather than a per-patient vector.

### 2.2. Btm: A Biterm Topic Model

**Figure 1:**
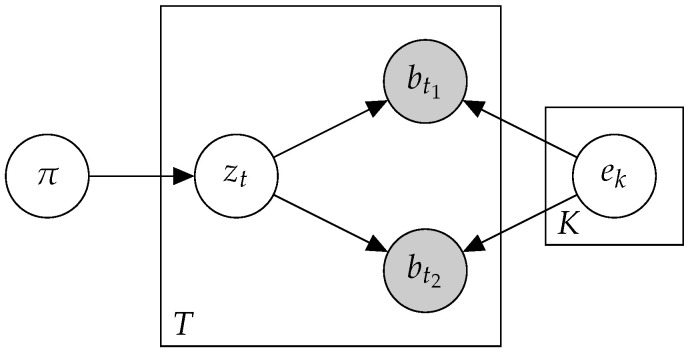
A plate diagram for Btm.

To enrich the input data, we adapted a method previously used to analyze short texts in [27]. Instead of viewing mutations as individuals, we examined their co-occurrence patterns with other mutations. Let a biterm be a pair of mutations that co-occur in the same patient. The assumption in Btm was that each biterm was the product of a single mutational process. Formally, patient *n* was determined by a sequence of biterms $\left(b_{1}^{n}\cdots b_{T_{n}}^{n}\right)$, where $b_{t}^{n}=b_{t_{1}}^{n},b_{t_{2}}^{n}$ and the corresponding mutations are represented by the hidden signature sequence $Z^{n}=\left(z_{1}^{n}\cdots z_{T_{n}}^{n}\right)$, as described in Figure 1.

Where $B^{n}\in N_{\geq 0}^{M\times M}$ is the biterm matrix for patient *n* and $B_{ij}^{n}=\left\{t_{1}\neq t_{2}|b_{t_{1}}=i,b_{t_{2}}=j\right\}$ is the number of times words *i* and *j* co-occur in the patient. Given the count vector $V_{n}$ of a patient, we constructed the biterm matrix as $B^{n}=V_{n}^{T}V_{n}-\mathrm{diag}\left(V_{n}\right)$. Given a high number of patients, we constructed the biterm matrix *B* as the summation of all the biterm matrices together:

$$B=\sum_{n=1}^{N}B^{n}=\sum_{n=1}^{N}V_{n}^{T}V_{n}-\mathrm{diag}\left(V_{n}\right)$$

Note that building *B*, at worst, cost $O(NM^{2})$, but it could also be calculated as O(|B|) if that was more efficient.We could also perform any combinations as required by the situation, i.e., for fewer patients with more than *M* mutations, we could use the matrix multiplication option, and for the rest, we computed biterms, one by one. We searched for $\pi =(\pi_{1}\cdots \pi_{K})$ and signatures *e*, as they could maximize the model’s success:

$$\begin{array}{cc} \; & \mathrm{Pr}\left[B|\pi ,e\right]=\prod_{i=1}^{M}\prod_{j=1}^{M}\mathrm{Pr}{\left[b=(i,j)|\pi ,e\right]}^{B_{ij}}\\ \; & =\prod_{i=1}^{M}\prod_{j=1}^{M}{\left(\sum_{k=1}^{N}\mathrm{Pr}\left[b=(i,j),z=k|\pi ,e\right]\right)}^{B_{ij}}\\ \; & =\prod_{i=1}^{M}\prod_{j=1}^{M}{\left(\sum_{k=1}^{N}\pi_{k}e_{k}\left(i\right)e_{k}\left(j\right)\right)}^{B_{ij}} \end{array}$$

We optimized the model using the following EM algorithm:

*E-step*: Compute for every i,j,k:

-
$p_{k|ij}=\mathrm{Pr}\left[z=k|b=(i,j),\pi ,e\right]=\frac{\pi_{k}e_{k}\left(i\right)e_{k}\left(j\right)}{\sum_{k^{\prime }=1}^{K}\pi_{k^{\prime }}e_{k^{\prime }}\left(i\right)e_{k^{\prime }}\left(j\right)}$

-
$E_{k}\left(i\right)=\sum_{j=1}^{M}B_{ij}p_{k|ji}+B_{ji}p_{k|ij}$

-
$A_{k}=\sum_{i=1}^{M}E_{k}\left(i\right)$

*M-step*: Compute for every i,k:

-
$\pi_{k}=\frac{A_{k}}{\sum_{k^{\prime }=1}^{K}A_{k^{\prime }}}$

-
$e_{k}\left(i\right)=\frac{E_{k}\left(i\right)}{\sum_{i^{\prime }=1}^{M}E_{k}\left(i^{\prime }\right)}$

Each EM iteration could be completed in $O(KM^{2})$ time for *K* signatures and *M* mutation categories. To avoid bad local minima, Btm was trained for 100 iterations from 10 random seeds, and then the best one was chosen and further trained for 500 additional iterations.

### 2.3. Mix: A Mixture of MMMs

For completeness, we briefly present the Mix method, which was previously developed in [25].

**Figure 2:**
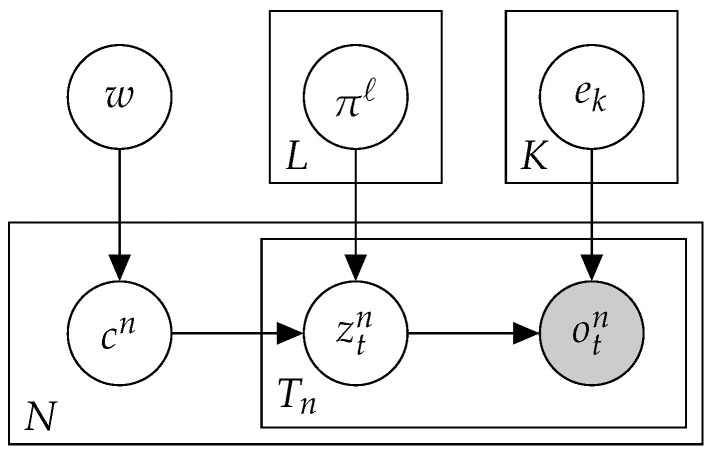
A plate diagram for Mix.

In order to handle sparse data, the Mix approach clustered the samples and learned the exposures per cluster, rather than per sample. To this end, we proposed a mixture model, which led to simultaneous optimizations of sample (soft) clustering, exposures, and signatures (Figure 2). Given the hyper-parameter *L*, which indicated the number of clusters, denoted by $c^{n}\in \left\{1\cdots L\right\}$, the hidden variables representing the true cluster identity of each sample. Our goal was to learn the cluster a priori probabilities $w=(w_{1}\cdots w_{L})$, cluster exposures $\pi =(\pi^{1}\cdots \pi^{L})$, and shared signatures *e*, so as to maximize the model’s success:

$$\begin{array}{cc} \; & \mathrm{Pr}\left[V|w,\pi ,e\right]=\prod_{n=1}^{N}\mathrm{Pr}\left[V^{n}|w,\pi ,e\right]=\prod_{n=1}^{N}\sum_{\ell =1}^{L}\mathrm{Pr}\left[c^{n}=\ell ,V^{n}|w,\pi ,e\right]\\ \; & =\prod_{n=1}^{N}\sum_{\ell =1}^{L}\mathrm{Pr}\left[c^{n}=\ell \right]\mathrm{Pr}\left[V^{n}|\pi^{\ell },e\right]=\prod_{n=1}^{N}\sum_{\ell =1}^{L}w_{\ell }\prod_{j=1}^{M}{\left(\sum_{i=1}^{K}\pi_{i}e_{i}\left(j\right)\right)}^{V_{j}} \end{array}$$

Similarly to Btm, the Mix model was optimized with an EM algorithm. Each iteration could be completed in O(NLKM). To avoid bad local minima, the Mix method was trained for 100 iterations from 10 random seeds, then the best one was chosen and further trained for 500 additional iterations.

### 2.4. Btm2K-Learning the Number of Signatures in a Dataset Using Btm

We present below a method to learn the hyper-parameter *K*, which was the number of signatures that underpinned a highly sparse dataset. Given a mutation matrix *V*, we applied a 2-fold cross-validation, training Btm with a varying number of signatures on one-half and testing the overlooked log-likelihood on the other, and vice versa. We repeated this process *T* times and chose the number of signatures with the best median overlooked log-likelihoods.Following the previous work [28], we further applied a rollback mechanism to choose the more concise solution in cases where the differences in log-likelihood were not significant.

Because the number of biterms was quadratic in the number of mutations in a given patient, small changes in the number of mutations could lead to larger changes in the number of biterms. To avoid this balancing problem in the cross-validation, we defined “big patients” as patients with more than 5 times the average number of biterms in the data. On all the datasets we tested, there were 1–3% big patients, containing 75–85% of the biterms. This phenomenon affected the cross-validation more than the number of signatures, and thus, we applied the cross-validation to the other patients only and used the big patients in addition to the training fold (i.e., they were used only for training alongside the training fold). The algorithm is summarized below. The method was summarized in the pseudo-code Algorithm 1.
**Algorithm 1**$\mathrm{Btm}2K(V,K_{\min},K_{\max})$.1:Input: $V\in R_{\geq 0}^{N\times M},1\leq K_{\min}<K_{\max}\leq \min\left\{N,M\right\}$2:Parameters: T=numberofrunsforeachK3:$V_{\mathrm{big}}=\mathrm{Samples}\;\mathrm{with}\;\mathrm{more}\;\mathrm{than}\;5\;\mathrm{times}\;\mathrm{the}\;\mathrm{average}\;\mathrm{biterms}\;\mathrm{in}\;V$4:V=Therest5:**for**t=1,⋯,T**do**6:     $V_{1},V_{2}=\mathrm{split}\;V\;\mathrm{randomly}\;\mathrm{to}\;\mathrm{two}\;\mathrm{equal}\;\mathrm{sized}\;\mathrm{sets}$7:     **for** $k=K_{\min},\cdots ,K_{\max}$ **do**8:          $\mathrm{btm}=\mathrm{BTM}(k,V_{1}⋃V_{\mathrm{big}})$9:          $S\left[k,t\right]=\mathrm{btm.log-likelihood}\left(V_{2}\right)$10:        $\mathrm{btm}=\mathrm{BTM}(k,V_{2}⋃V_{\mathrm{big}})$11:        $S\left[k,t\right]=S\left[k,t\right]+\mathrm{btm.log-likelihood}\left(V_{1}\right)$12:$\tilde{K}=\underset{k}{arg min}\left(\mathrm{median}\left(S\left[k,:\right]\right)\right)$13:**repeat**14:    $K^{*}=\tilde{K}$15:    $\tilde{K}=\min\left\{K<K^{*}|\mathrm{Wilcoxons-rank-sum}\left(S\left[K,:\right],S\left[K^{*},:\right]\right)>0.05\right\}$16:**until**$\tilde{K}<K^{*}$17:**return**$\tilde{K}$

### 2.5. Previous Hyper-Parameter Selection Algorithms

There were several previous algorithms for selecting the number of signatures in a dataset. For rich data, one of the leading methods, CV2K [28], was based on testing the ability of NMF to reconstruct overlooked data when varying its number of components (which corresponded to signatures).

For sparse data, the only previous method that was used in Mix was based on the Bayesian information criterion (BIC), which combines model likelihood with its number of parameters. In the case of Mix, the BIC was applied to select the number of signatures as well as the number of model clusters, thus requiring the model likelihood evaluation in settings with many parameters.

### 2.6. Running-Time Estimation

For simplicity, we assumed that each model required the same number of iterations *R* to converge and that BIC was iterated over all options for the number of clusters, from 1 to $L_{\max}$. To train a model, we used 10 random seeds and improved them for 100 iterations, and then chose the best one and trained it for 500 more iterations, so R=1500. We also assumed that Btm2K and CV2K were both processed T=30 times. Last, we iterated through all the options for $K=1\cdots K_{\max}$ signatures, denoted by N,M the number of samples and mutation categories (96), respectively. Then, the algorithms’ complexities were as follows (Table 1):

1. Btm2K: For a given *k*, we needed to train Btm 2T times (*T* repetitions of 2 folds). To train Btm, we needed to create biterms with $NM^{2}$ time and $RkM^{2}$ training time. In total the cost for *k* was $2TNM^{2}+2TRkM^{2}$. Note that we created biterms one time for all *k* in each run, so in total, the run time was $∼2TNM^{2}+TRK_{\max}^{2}M^{2}$.
2. BIC: For a given *k*, we considered all possible $L=1\cdots L_{\max}$. For a given pair, we trained Mix once for a cost of RNkLM. In total, for all *L*s, we needed $∼RkNML_{\max}^{2}/2$. Overall, $∼RK_{\max}^{2}L_{\max}^{2}NM/4$ was needed.
3. CV2K: For a given *k*, we needed to train NMF *T* times, and each iterations cost NkM time, for a total of TRNkM time. In total, for all *k*, we spent $TRNK_{\max}^{2}M$.

**Figure 3:**
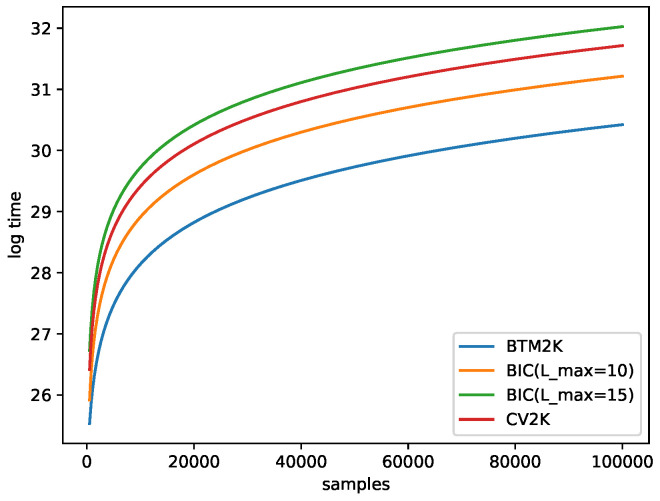
Log running time estimation for Btm2Kand BIC with a maximum of 10–15 clusters and CV2K as a function of the number of samples. Here, $K_{\max}=10$ was used. For CV2K and Btm2K, $L_{\max}=15$ was used.

Note that for Btm2K and CV2K, the cost did not include learning the number of clusters; thus, if we want to train Mix, we needed to use BIC and find the number of clusters. This added $∼RK_{\max}NML_{\max}^{2}/2$ more time to the process. Figure 3 shows that Btm2K was order of magnitudes faster than the other methods.

### 2.7. Data

We present below both real panel datasets, as well as down-sampled and simulated datasets on which we tested our model.

1. *MSK-IMPACT [20,22] Pan-Cancer.* We downloaded mutations for a cohort of patients from Memorial Sloan Kettering Integrated Mutation Profiling of Actionable Cancer Targets (MSK-IMPACT), which was targeted sequencing data from https://www.cbioportal.org/ (accesed on 1 January 2022). The MSK-IMPACT dataset contained 11,369 pan-cancer patients’ sequencing samples across 410 target genes. We restricted our analysis to 18 cancer types with more than 100 samples, which resulted in a dataset of 5931 samples and about 7 mutations per sample.
2. *Whole genome/exome (WGS/WXS) data*. We combined mutations from different sources and cancer types of whole-genome-sequencing and whole-exome-sequencing (WGS/WXS): ovarian cancer (OV), chronic lymphocytic leukemia (CLL), malignant lymphoma (MALY), and colon adenocarcinoma (COAD). We downloaded the OV samples from the Cancer Genome Atlas [29]. For CLL and MALY, we used ICGC release 27, analyzed the sample with the most mutations per patient, and restricted those to samples annotated as “study = PCAWG” [24]. For evaluation purposes, we down-sampled the data to target regions of MSK-IMPACT [20,22]. The data characteristics are summarized in Table 2.
3. *Simulated data.* The simulated data were generated and described in detail in [16] to evaluate SigProfiler (SP) and SignatureAnalyzer (SA). Here, for each of the 12 datasets, we evaluated our method on two sets of realistic synthetic data: SP-realistic, based on SP’s reference signatures and attributes, and SA-realistic, based on SA’s reference signatures and attributes. For each of the (i)–(x) tests, the synthetic datasets were generated based on observed statistics for each signature of each cancer type. Different datasets could differ by the number of signatures, the number of active signatures per samples (sparsity), the number of mutations per sample (whole exome/genome sequencing), whether they reflected a single cancer type or multiple types, and the similarity between signatures. All these factors affected the difficulty of determining the number of components. For each simulated sample, we sampled an MSK-IMPACT patient and down-sampled the simulated sample, so it had the same number of mutations. We removed datasets with missing mutation categories.

### 2.8. Implementation Details

Btm was implemented in Python 3 using numpy [30]. For NMF, we used the scikit-learn implementation [31]. The code for Mix was available at https://github.com/itaysason/Mix-MMM (accesed on 1 January 2022), and the code for CV2K was sourced from https://github.com/GalGilad/CV2K/ (accesed on 1 January 2022).

## 3. Results

### 3.1. Evaluating the Number of Signatures from Simulated Data

We applied Btm2K to a range of datasets to test its performance and compare the results to current methods. In our first set of results, we used a down-sampled version of the simulated data from [16]. While each dataset was generated by a known set of signatures, due to the down-sampling, this true number may not be reflected in the remaining mutations, which was potentially a result of having only a subset of the true signatures. To mitigate this difficulty, we matched each mutation to a signature with maximum a priori probability (using the known exposures and known signatures).Next, we counted the occurrences of each signature in the down-sampled sample and summed all samples in the dataset. We reported the number of signatures that appeared in more than 5% of the mutations in the down-sampled data. We omitted datasets where all the methods inferred a single signature. The results are summarized in Table 3 and show the superiority of Btm2K over the other approaches.

### 3.2. Evaluating the Number of Signatures from MSK-IMPACT Data

**Figure 4:**
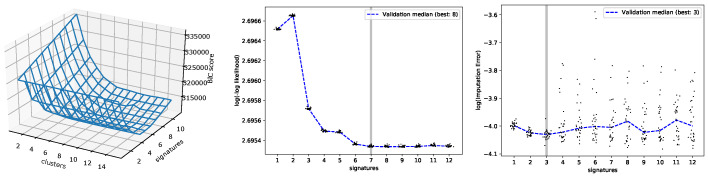
Performance evaluation on MSK-IMPACT data for varying number of signatures. **Left**: BIC scores of Mix with varying parameters. **Middle**: Btm2K log of minus log-likelihoods. **Right**: CV2K log reconstruction errors. For the middle and right panels, dots represent runs with their median denoted by a dashed line. The minimum median is denoted in the figure legend and the final chosen K is marked in gray.

Next, we applied Btm to analyze 5931 samples from the MSK-IMPACT dataset. In Figure 4, the performances of the three estimation methods on this dataset are shown. BIC, Btm2K, and CV2K estimated 6, 7, and 3 signatures, respectively. BIC took around 100 hours to learn both parameters while Btm2K took 1 hour to learn the number of signatures (BIC required 8 additional hours to learn the number of clusters). Complexity-wise, Btm2K was 10–100-fold faster than BIC. To evaluate the quality of their estimations, we trained Mix, Btm, and NMF models on 3, 6, and 7 signatures, respectively, and then we assessed the quality of signatures and log-likelihood of the resulting model on unseen data. We presented the results in the range of 3–9 signatures.

**Figure 5:**
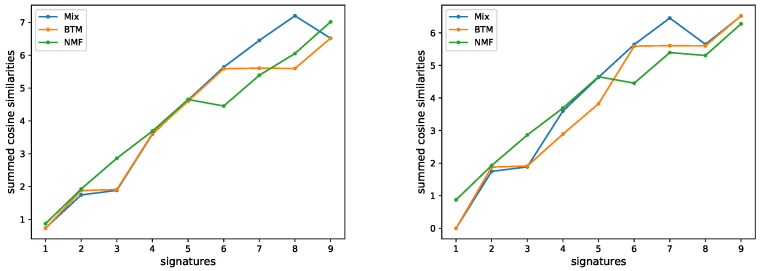
Summed cosine similarities of de novo signatures and COSMIC signatures. In the summation, only unique signatures with similarity above 0.7 (**left**) or 0.8 (**right**) were considered.

**Figure 6:**
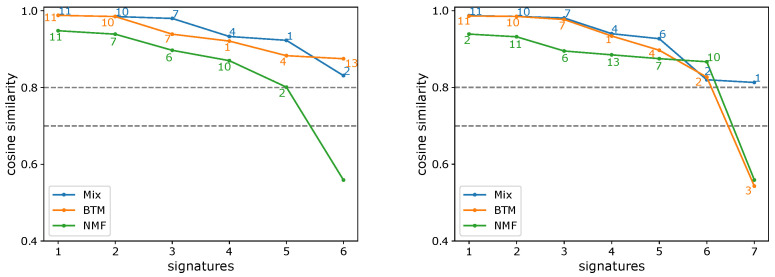
De novo signature discovery of MSK-IMPACT panel data. Shown are the sorted cosine similarities between learned signatures and most similar COSMIC signature (denoted next to the plot) for Mix, Btm, and NMF, across a range of number of signatures (6, 7 corresponding to (**left**) and (**right**), respectively). Repeating signatures of the same model are not shown.

To evaluate the quality of the learned signatures, we compared them to the COSMIC signatures. We matched each learned signature to the most similar COSMIC signatures (cosine similarity). We used 0.7 and 0.8 thresholds to determine if a signature was similar to a COSMIC signature. If two signatures were similar to the same COSMIC signature, we determined that the signature with the lower similarity was a duplicate. The results are summarized in Figure 5 and showed that for both thresholds, the maximum number of high quality signatures that had been learned was 7, supporting the estimate of Btm2K and suggesting the other methods underestimated the true number. A more detailed view of the learned signatures appears in Figure 6. Evidently, Btm learned high-quality signatures at a fraction of the time Mix used, supporting its improvements.

**Figure 7:**
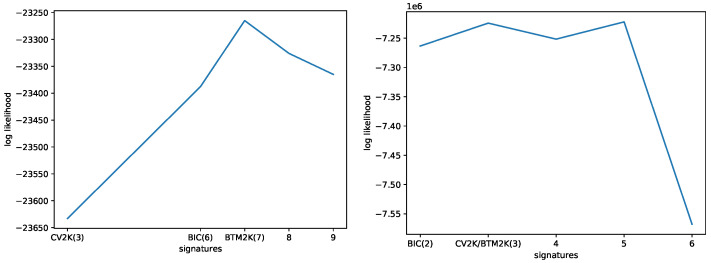
Log-likelihood of Mix on unseen data as a function of the number of signatures. **Left**: Mix was trained on MSK-IMPACT data and tested on the down-sampled WGS/WXS data. **Right**: Mix was trained on down-sampled WGS/WXS data and tested on the original data.

To further show the advantage of the Btm-inspired method Btm2K, we used Mix to compute the likelihood of yet unseen down-sampled WGS/WXS data, with the different numbers of signatures. For each number of signatures chosen, we used BIC to learn the best number of clusters. The results appear in Figure 7 (left panel) and show that seven signatures, as suggested by Btm2K  outperformed the other choices. Of interest, eight signatures performed worse than seven signatures, supporting the use of the rollback mechanism in Btm2Kto avoid over-fitting.

Last, we used the three methods to estimate the number of signatures on the down-sampled data. The methods estimated 2 (BIC) and 3 (Btm2K and CV2K) signatures. We trained Mix with these parameters and estimated the performance on the full WXS/WGS mutation catalogs. As shown in Figure 7 (right panel), although the five signatures performed better than three, the latter outperformed the BIC choice of two signatures.

## 4. Conclusions

We adapted Btm, which was developed for the task of handling short texts, and showed it to be useful on panel mutation data. We then developed Btm2K, a method that used Btm to select the number of components on sparse data, such as panel mutations. Our method performed well on several real and simulated datasets, with considerable computational benefits, as compared to current methods. A particularly interesting use case for this method was as a pre-processing step for Mix  serving as a better and faster way to choose hyper-parameters. Future work should harness this approach to learn improved topic models for sparse mutation data.

## Figures and Tables

**Table 1:** Summary of time complexity for BIC, Btm2K, and CV2K. Here, R,N, and *M* denotes number of iterations to train a model (1500), samples, and categories (96). *T* denotes the number of repetitions of Btm2K and CV2K (30), and $K_{\max}$ $L_{\max}$ denotes the maximum number of signatures and clusters used when the methods iterated.

Method	∼Learning Number of Signatures Complexity	∼Learning Number of Clusters Complexity (BIC)
BIC	$RK_{\max}^{2}L_{\max}^{2}NM/4$	
Btm2K	$2TNM^{2}+TRK_{\max}^{2}M^{2}$	$RK_{\max}NML_{\max}^{2}/2$
CV2K	$TRNK_{\max}^{2}M$	$RK_{\max}NML_{\max}^{2}/2$

**Table 2:** Summary of WGS/WXS down-sampled datasets.

Cancer	#Samples	#Mutations	#Panel Mutations
OV	411	46,299	1812
Maly	100	1,220,526	1770
CLL	100	270,870	278
COAD	44	52,827	1789
Combined	653	1,590,520	5604

**Table 3:** Estimation of number of signatures in simulated data. For Btm2K and CV2K, the numbers of the best run and the numbers after rollback are shown. In the last column, the number of signatures were present in more than 5% of the mutations as an estimate for the true solution. In bold are the methods that performed best with regard to this estimate.

Data Set	BIC	Btm2K	CV2K	# Signatures with >=5% Down-sampled Mutations
ii-sa	3	**4->4**	4->2	8
ii-sp	3	**10->7**	4->2	6
v-sa	2	**3->3**	3->2	6
v-sp	2	**3->2**	**6->2**	5
vii.a(pri.)-sp	1	**2->2**	3->1	2
vii.b(sec.)-sa	1	1->1	**5->2**	3
viii-sp	1	**2->2**	5->1	7
ix-sa	2	**4->4**	4->2	8
ix-sp	4	**6->6**	4->3	6
x-sa	1	**3->3**	5->1	8
x-sp	1	**6->6**	5->4	6

## Data Availability

The data presented in this study are available on request from the corresponding author.
